# Filamentous Fungi and the Biodeterioration of Organic Cultural Heritage Materials: A Systematic Review of Mechanisms, Risks, and Preventive Conservation Strategies

**DOI:** 10.3390/microorganisms14030526

**Published:** 2026-02-25

**Authors:** Giancarlo Angeles Flores, Roberto Venanzoni, Sabata Martino, Paola Angelini

**Affiliations:** 1 Department of Chemistry, Biology and Biotechnology, University of Perugia, Via del Giochetto, 06122 Perugia, Italy; giancarlo.angelesflores@unipg.it (G.A.F.); roberto.venanzoni@unipg.it (R.V.); sabata.martino@unipg.it (S.M.); 2 Centro di Ricerca per l’Innovazione, Digitalizzazione, Valorizzazione e Fruizione del Patrimonio Culturale e Ambientale (CE.D.I.PA.), Piazza San Gabriele dell’Addolorata, 4, 06049 Spoleto, Italy

**Keywords:** cellulose-based heritage materials, climate change impacts, enzymatic degradation, fungal colonization, indoor environments, systematic review, microclimate instability, preventive conservation, stress-tolerant fungi

## Abstract

Filamentous fungi are among the most significant biological agents responsible for the biodeterioration of organic cultural heritage materials preserved in archives, libraries, and museums. Cellulose-based substrates—such as paper, papyri, and plant-derived textiles—as well as protein-based materials, including parchment and leather, provide favourable conditions for fungal colonization due to their chemical composition and hygroscopic behaviour. Once activated, fungi contribute to deterioration through a combination of mechanical penetration and biochemical processes, including the secretion of hydrolytic enzymes, organic acids, and pigmented metabolites, which progressively compromise the structural integrity and visual appearance of heritage objects. This review aims to critically synthesize current knowledge on the mechanisms of fungal biodeterioration affecting organic heritage materials, with particular attention to material-specific vulnerabilities, indoor environmental drivers, and implications for preventive conservation. Recent advances in fungal ecology have highlighted the presence of xerophilic and extremotolerant taxa capable of persisting under conditions traditionally considered unfavourable for microbial growth, posing new challenges for conservation management. Rather than attributing biodeterioration directly to global climate change, this review explicitly emphasizes the role of indirect and building-mediated climate-related stressors—such as increased frequency of moisture intrusion events, infrastructure vulnerability, and microclimatic instability within buildings—in shaping fungal risk in indoor heritage environments. The integration of environmental monitoring, microbiological diagnostics, and predictive risk-assessment tools emerges as a key strategy for early detection and mitigation. By consolidating interdisciplinary evidence from microbiology, materials science, and heritage conservation, this work underscores the need to shift from reactive restoration toward anticipatory, risk-based preventive approaches to ensure the long-term preservation of organic cultural heritage materials.

## 1. Introduction

Fungal biodeterioration represents one of the most pervasive biological threats to organic cultural heritage materials preserved in archives, libraries, and museums [1,2,3]. A wide range of heritage objects—including manuscripts, books, archival documents, papyri, parchment artefacts, textiles, and canvas supports—are inherently susceptible to microbial colonization due to their organic composition and long-term exposure to indoor environments [4]. Among the diverse microbial groups encountered in heritage contexts, filamentous fungi play a particularly critical role owing to their ecological ubiquity, efficient dispersal through airborne spores, and pronounced capacity to exploit low-nutrient substrates [5].

Organic heritage materials can be broadly categorized according to their primary chemical composition. Cellulose-based substrates, such as paper, papyri, and plant-derived textiles, are especially vulnerable to fungal attack because cellulose serves as a readily accessible carbon source for cellulolytic microorganisms [6]. In contrast, protein-based materials—including parchment and leather, which are primarily composed of collagen—are preferentially colonized by proteolytic fungi capable of degrading proteinaceous matrices [7]. In addition, many heritage objects, such as canvas paintings and composite artefacts, are inherently polymaterial systems in which organic supports coexist with preparatory layers, binders, pigments, and structural components. This material heterogeneity creates complex microenvironments that can further enhance fungal persistence and biodeterioration processes [8].

Once established on heritage substrates, filamentous fungi contribute to deterioration through a combination of mechanical and biochemical mechanisms. Hyphal penetration into fibrous matrices disrupts structural cohesion, while the secretion of extracellular hydrolytic enzymes—such as cellulases, hemicellulases, and proteases—leads to progressive depolymerization of cellulose and collagen. These processes are frequently accompanied by the production of organic acids and pigmented secondary metabolites, which promote local acidification, chromatic alteration, and destabilization of inks, sizing agents, and binding media [9]. Importantly, fungal activity may remain subclinical for extended periods, producing significant chemical and mechanical weakening before visible signs of damage become apparent.

The development and progression of fungal biodeterioration in heritage environments are strongly governed by indoor microclimatic conditions. Relative humidity, temperature, water activity, and air circulation patterns exert a decisive influence on spore germination, mycelial growth, and enzymatic activity [3,10]. Because organic heritage materials are hygroscopic, even moderate fluctuations in environmental parameters can create localized conditions favourable to fungal activation. While preventive conservation strategies are designed to maintain indoor climates below critical biological thresholds, recent studies have documented the presence of xerophilic and extremotolerant fungal taxa capable of persisting under relatively dry or fluctuating conditions, thereby challenging traditional assumptions of microbial control [5].

In this context, fungal biodeterioration should not be interpreted as a direct consequence of global climate change acting uniformly on heritage collections. Rather, climate-related risks manifest predominantly through indirect pathways, including increased frequency of moisture intrusion events, infrastructure vulnerability, HVAC malfunction, and microclimatic instability within buildings [10]. These factors can compromise the effectiveness of preventive measures and create episodic conditions conducive to fungal outbreaks, particularly in historic structures with limited environmental buffering capacity.

The aim of this review is to provide a critical and integrative synthesis of current knowledge on the biodeterioration of organic cultural heritage materials by filamentous fungi. Specifically, the review examines (i) the material-specific vulnerabilities of cellulose-based, protein-based, and polymaterial substrates; (ii) the principal fungal taxa and mechanisms involved in biodeterioration; (iii) the role of indoor environmental drivers in modulating fungal risk; and (iv) the implications for preventive conservation and risk-based management. By consolidating evidence across microbiology, materials science, and heritage conservation, this work seeks to support the development of anticipatory, scientifically grounded strategies for the long-term preservation of organic cultural heritage.

## 2. Materials and Methods

This review was conducted following general principles of transparent and reproducible literature selection and synthesis, in line with PRISMA 2020 recommendations [11]. Given the interdisciplinary and heterogeneous nature of research on fungal biodeterioration of cultural heritage materials, a narrative synthesis approach was adopted rather than a quantitative meta-analysis. In this review, “biodegradation” refers to the enzymatic breakdown of organic polymers at the molecular level, whereas “biodeterioration” denotes the broader heritage-relevant outcome of biological activity, including structural, aesthetic, and informational damage to cultural materials.

### 2.1. Literature Search Strategy

A comprehensive literature search was carried out between January and February 2025 using Scopus, Web of Science Core Collection, and PubMed. These databases were selected to retrieve literature spanning heritage science, microbiology, and indoor environmental research. PubMed was used as a supplementary source to retrieve studies addressing fungal ecology and biodeterioration mechanisms relevant to indoor heritage environments.

Search queries combined free-text keywords related to filamentous fungi, biodeterioration, organic cultural heritage materials, preventive conservation, and indoor microclimatic conditions. The literature search was performed using combinations of keywords related to filamentous fungi, biodeterioration, organic cultural heritage materials, preventive conservation, and indoor environments. Google Scholar was used selectively to identify additional relevant publications and to verify citation completeness.

Only peer-reviewed articles published in English between 2010 and 2025 were considered. Grey literature and non–peer-reviewed sources were excluded.

### 2.2. Eligibility Criteria and Study Selection

Study eligibility was defined according to the PICOS framework [12]. Included studies were required to focus on organic cultural heritage materials, encompassing cellulose-based, protein-based, or polymaterial substrates [13], to investigate filamentous fungi or fungal communities associated with biodeterioration [14], and to report information on deterioration mechanisms, environmental drivers, diagnostic approaches, or conservation implications [15].

**Figure 1:**
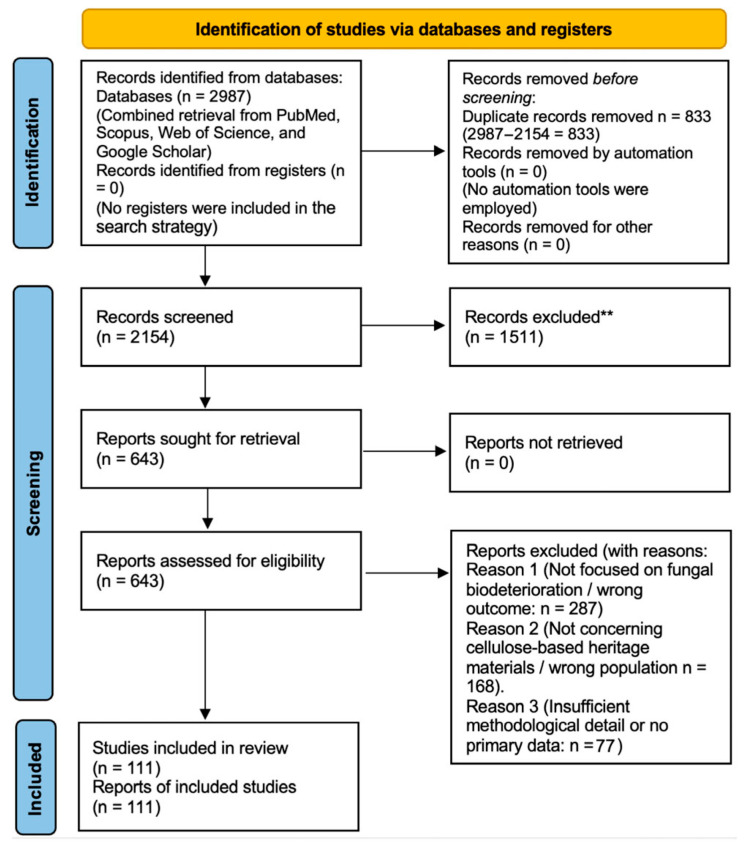
PRISMA 2020 flow diagram showing the study selection process, including records identified, duplicates removed, studies screened, full-text assessments, exclusions with reasons, and the final number of studies included in the review.

After automated and manual deduplication, titles and abstracts were screened independently by two authors, followed by full-text assessment of potentially relevant articles [16]. Any disagreements were resolved through discussion until consensus was reached. The study selection process is summarized in Figure 1.

### 2.3. Data Extraction and Synthesis

For each included study, relevant information was extracted concerning the type of heritage substrate, fungal taxa and ecological traits, analytical and diagnostic methods, reported environmental conditions, and documented deterioration patterns or conservation implications [17,18,19,20,21].

Due to the diversity of materials, methodologies, and outcome measures across studies, a quantitative synthesis was not feasible. The results were therefore synthesized narratively, grouping evidence thematically according to material type, fungal mechanisms of biodeterioration, environmental drivers, and preventive conservation strategies.

This approach allowed the identification of recurring trends, convergent findings, and material-specific vulnerabilities relevant to risk-based conservation management [22,23].

### 2.4. Registration and PRISMA 2020 Compliance

This systematic review was conducted and reported in accordance with the PRISMA 2020 (Preferred Reporting Items for Systematic Reviews and Meta-Analyses) guidelines. The PRISMA 2020 checklist and flow diagram were used to guide study identification, screening, eligibility assessment, and inclusion.

The review protocol was not registered in an international prospective registry (e.g., PROSPERO), as the study focuses on environmental microbiology and cultural heritage conservation rather than clinical or health-related outcomes.

Nevertheless, the methodology, eligibility criteria, and study selection process were defined a priori and are transparently reported to ensure reproducibility and methodological rigor.

## 3. Indoor Environments

Fungal biodeterioration of organic cultural heritage materials occurs predominantly within indoor environments such as archives, libraries, museums, and storage facilities, where microclimatic conditions play a decisive role in regulating microbial activity [23,24,25]. Unlike outdoor settings, indoor heritage environments are characterized by controlled—but often imperfectly stable—environmental parameters, which can create localized niches favourable to fungal survival and growth [26].

Among the environmental variables influencing fungal biodeterioration, relative humidity represents the primary controlling factor [27,28,29]. Elevated humidity levels promote spore germination, hyphal growth, and enzymatic activity, while fluctuations around critical thresholds can induce repeated cycles of activation and dormancy. Because cellulose-based and protein-based heritage materials are hygroscopic, they readily absorb and release moisture in response to ambient conditions, generating microenvironments at the material surface that may differ substantially from bulk air measurements [30,31]. Consequently, fungal development may occur even when average room conditions appear to fall within recommended conservation ranges [32].

Temperature acts as a secondary but synergistic factor, modulating metabolic rates and influencing fungal community composition [33,34]. Within the temperature ranges typically encountered in indoor heritage environments, temperature alone rarely limits fungal growth; rather, its effects are closely coupled with moisture availability. Moderate increases in temperature can exacerbate biodeterioration processes when accompanied by elevated relative humidity or condensation events, particularly in poorly ventilated spaces [35].

Air circulation and ventilation patterns further influence fungal biodeterioration by affecting moisture distribution, dust accumulation, and spore dispersal [36]. Inadequate air exchange may lead to stagnant microclimates, promoting moisture retention and the persistence of fungal propagules on material surfaces. Conversely, excessive air movement can facilitate the redistribution of spores within storage areas, increasing the risk of cross-contamination between objects [37]. These dynamics underscore the importance of balanced ventilation strategies tailored to the specific characteristics of heritage spaces.

Light exposure generally plays a limited role in fungal biodeterioration within storage environments, as many collections are maintained under low-light conditions [38]. However, localized light sources may contribute indirectly by influencing surface temperature and microclimatic gradients, particularly in display contexts. In contrast, particulate matter and settled dust represent significant but often underestimated cofactors. Dust can act both as a nutrient reservoir and as a vector for fungal spores, enhancing colonization potential when combined with favourable moisture conditions [39].

Recent studies have highlighted the ability of certain xerophilic and extremotolerant fungal taxa to persist under relatively dry or fluctuating indoor conditions traditionally considered unfavourable for microbial growth [40]. The presence of these organisms does not invalidate preventive conservation strategies but rather emphasizes the need to interpret environmental thresholds as probabilistic risk indicators rather than absolute guarantees of safety.

Taken together, these factors demonstrate that fungal biodeterioration in indoor heritage environments arises from the interaction of multiple environmental drivers rather than from single parameters acting in isolation [41].

Effective preventive conservation therefore requires integrated environmental monitoring approaches capable of capturing microclimatic variability at the object level, combined with adaptive management strategies that account for material-specific vulnerabilities and building-related constraints.

## 4. Writing Supports (Cellulose-Based)

Filamentous fungi represent the most pervasive and destructive biodeteriogens affecting organic cultural heritage materials [42]. Their ecological ubiquity, combined with an exceptional capacity to exploit nutrient-poor substrates, enables them to colonize a wide variety of cellulosic and proteinaceous supports typically found in archives, libraries and museums [43,44,45]. Taxa belonging to the genera *Aspergillus*, *Penicillium*, *Cladosporium* and *Chaetomium* are consistently detected across heritage collections, where they initiate complex deterioration processes driven by synergistic biochemical and mechanico-physical mechanisms [46,47,48].

The biodeteriorative activity of filamentous fungi relies primarily on the secretion of extracellular hydrolytic enzymes—including cellulases, hemicellulases, pectinases and proteases—capable of depolymerizing cellulose and collagen matrices, thereby undermining fibre cohesion and structural stability [48,49]. Concurrently, many species produce melanin pigments and low-molecular-weight metabolites such as organic acids and oxidative agents, which contribute to acidification phenomena, chromatic staining, and destabilisation of sensitive components such as inks, sizing agents and binding media [50,51,52].

**Figure 2:**
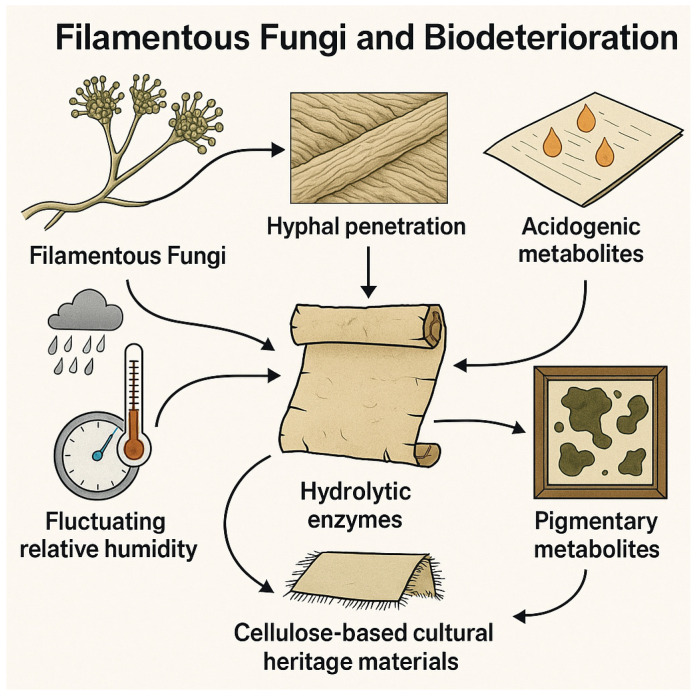
Overview of the main mechanisms by which filamentous fungi deteriorate cellulose-based cultural heritage materials, including hyphal penetration, hydrolytic enzyme secretion, and acidogenic or pigmentary metabolite production. These processes are intensified by environmental instability—especially fluctuations in relative humidity—leading to accelerated structural and chromatic degradation.

From a physiological standpoint, biodeteriogenic fungi exhibit a suite of intrinsic adaptations that enhance their colonization potential. These include the ability to thrive under oligotrophic conditions, the production of highly resistant spores, metabolic plasticity allowing modulation of enzymatic profiles in response to substrate chemistry, and active mechanical penetration of fibrous microstructures through turgor-driven hyphal force combined with enzymatic weakening of cell wall components [47,50,51] (Figure 2). Collectively, these characteristics explain the resilience and persistence of fungal contamination even in controlled indoor environments.

### 4.1. Material-Specific Vulnerabilities

The susceptibility of organic heritage materials to fungal attack is closely linked to their chemical composition, hygroscopic behaviour and microstructural architecture, all of which influence species selectivity and the predominance of particular degradation pathways (Table 1).

#### 4.1.1. Paper and Papyrus (Cellulosic Substrates)

Paper and papyrus, being rich in cellulose, are especially vulnerable to cellulolytic fungi such as *Chaetomium globosum*, *Trichoderma* spp. and various *Aspergillus* species, whose enzymatic complexes induce fibre fragmentation, loss of mechanical strength and progressive surface erosion [49,52].

Ancient papyri often contain residual plant polysaccharides, mineral inclusions and salts, which may favour colonization by halotolerant or microtolerant taxa adapted to fluctuating moisture regimes [52].

#### 4.1.2. Parchment and Leather (Protein-Based Substrate)

Parchment and leather, composed predominantly of denatured collagen, are preferentially degraded by proteolytic fungi—including *Aspergillus*, *Eurotium* and *Engyodontium* spp.—whose proteases weaken collagen fibres, resulting in gelatinization, surface detachment, curling and loss of elasticity [53].

Their marked hygroscopicity further amplifies susceptibility during episodes of elevated moisture.

#### 4.1.3. Canvas and Textile Supports

Canvas supports composed of linen or cotton fibres exhibit degradation patterns similar to those of paper, yet the presence of oils, binders, adhesives and organic pigments provides additional nutrient reservoirs.

Fungi such as *Cladosporium* and *Paecilomyces* are capable of penetrating deeply into textile weaves, while pigmented species produce persistent chromatic alterations that are often resistant to restoration interventions [54,55].

### 4.2. Intrinsic Adaptations and Mechanisms of Fungal Persistence

Biodeteriogenic fungi exhibit a remarkable suite of intrinsic physiological traits that substantially enhance both their survival and their degradative capacity on heritage substrates. Many species demonstrate pronounced oligotrophic resilience, enabling growth under conditions of extreme nutrient scarcity—an ecological strategy exemplified by indoor-adapted taxa such as *Aspergillus restrictus* and *Wallemia sebi* [9,56]. Their ability to produce highly stress-resistant spores further supports long-term persistence, as these propagules withstand prolonged desiccation, thermal oscillations and UV exposure, remaining viable until microenvironmental conditions become favourable for germination [47].

Equally significant is their metabolic and enzymatic plasticity, which allows fungi to modulate the secretion of hydrolytic enzymes in accordance with the polymeric composition of the substrate, thereby optimising degradative performance [48]. Many biodeteriogenic species also undergo melanogenesis, a process that affords protection against oxidative stress while simultaneously generating dark, persistent stains on sensitive surfaces [49,50,51]. Finally, turgor-driven hyphal penetration enables these organisms to infiltrate microfissures and deeper structural layers of materials by coupling mechanical force with the progressive enzymatic softening of fibres. Together, these adaptations firmly establish filamentous fungi as exceptionally efficient biodeteriogeni, capable of initiating subtle and often subclinical deterioration that precedes any visible manifestation of damage.

## 5. Environmental Instability and Climate-Related Risk for Indoor Heritage

Environmental parameters constitute the fundamental determinants of fungal colonisation dynamics on cultural heritage materials. Temperature, relative humidity (RH), water activity (aw) and indoor air quality regulate the transition from spore dormancy to active mycelial proliferation, shaping both the enzymatic activity of biodeteriogenic fungi and the composition of microbial communities colonising cellulose- and collagen-based substrates [57].

Due to the intrinsic hygroscopic behaviour of paper, parchment and painted surfaces, these materials undergo continuous moisture exchange with the surrounding air; recurring RH fluctuations therefore induce mechanical stress, microfracturing and localised softening that significantly facilitate hyphal penetration and fibre exploitation [58].

Critical moisture thresholds for fungal activation are well established. Most cellulolytic fungi become metabolically active above 65% RH, while enzymatic kinetics increase markedly between 80% and 95% RH [59].

Within a risk-based preventive conservation framework, international standards and institutional guidelines provide an essential methodological reference for the definition of acceptable environmental thresholds and management priorities. In particular, ISO standards for environmental monitoring and risk management, together with IFLA guidelines for library and archival environments, offer structured criteria for the systematic identification and assessment of biological and microclimatic risks affecting heritage materials. Their analytical integration supports evidence-based decision-making and facilitates the translation of monitoring data into preventive actions rather than reactive interventions.

Even brief condensation events—particularly on cold surfaces, in poorly ventilated areas or within stratified layers of composite materials—may trigger spore germination under otherwise acceptable indoor conditions [60]. Indoor aerobiology further contributes to biodeterioration risk: high airborne concentrations of *Aspergillus*, *Penicillium* and *Cladosporium*, often recirculated through HVAC systems or resuspended by human activity, provide a persistent inoculum capable of initiating colonisation when microclimatic conditions become favourable [61]. Dust deposition acts both as a reservoir of spores and as an auxiliary nutrient source, as its organic fraction supports rapid fungal activation following transient humidification [62].

Within this framework, climate change should not be regarded as a direct cause of fungal biodeterioration, but rather as a factor that amplifies environmental instability in indoor heritage settings. Prolonged heatwaves, intensified storm events and the increasing frequency of water ingress or HVAC malfunction promote conditions that favour thermotolerant and xerophilic fungi, taxa that were previously considered marginal in heritage environments [63]. Global warming and erratic humidity cycles destabilise indoor microclimates, reducing the effectiveness of conventional preventive conservation strategies and increasing the likelihood of episodic fungal activation [63]. In this evolving scenario, early-warning environmental monitoring and adaptive climate-management systems become essential tools for safeguarding organic collections.

### 5.1. Water Activity and Microclimate Modeling

Water activity (aw) represents a more precise indicator than RH for assessing microbial growth potential, as it reflects the bioavailable fraction of moisture required for metabolic processes. Most cellulolytic fungi activate at aw ≥ 0.70, whereas xerophilic species may thrive at values as low as 0.65 [64]. Localised aw hotspots—such as those found within layered structures, inside bindings, or beneath glazing systems—often elude detection by room-level dataloggers, creating concealed niches of heightened fungal risk.

To address these challenges, computational microclimate modelling and multi-point environmental sensing have become essential components of modern preventive conservation frameworks. These approaches enable the identification of enclosed zones prone to moisture accumulation, including sealed frames, display cases and storage enclosures [65]. Moreover, predictive models built upon material-specific sorption isotherms allow conservators to anticipate the hygroscopic responses of substrates such as papyrus and parchment, providing a more accurate assessment of vulnerability profiles under increasingly variable climatic conditions [66]. At the interface between environmental monitoring and risk assessment, it is useful to define approximate thermo-hygrometric thresholds beyond which fungal activation and damage become increasingly likely.

Table 2 summarises the main critical climate thresholds for fungal biodeterioration of organic heritage materials, highlighting their implications for preventive conservation.

### 5.2. HVAC Design and Filtration Standards

HVAC systems play a central role in controlling indoor microclimatic stability, airborne particulate matter and fungal propagule dispersion in heritage environments [67]. When properly designed and maintained, they contribute to maintaining thermo-hygrometric conditions below biological activation thresholds. Conversely, inadequate filtration, unbalanced airflow or system malfunction can exacerbate microbial contamination and moisture instability, increasing the risk of fungal biodeterioration [68].

Filtration efficiency is a critical parameter. Insufficient filters allow continuous circulation of airborne conidia, particularly those of *Aspergillus* and *Penicillium*, which may be redistributed throughout storage and exhibition spaces via ventilation pathways [67]. International conservation and HVAC guidelines therefore recommend the adoption of high-efficiency filtration systems, such as HEPA or MERV 13–16 filters, to effectively reduce fungal spore loads in museums, libraries and archives [67].

Airflow patterns and ventilation design further influence fungal risk. Poorly ventilated zones, stagnant air pockets and areas shielded from airflow—such as behind shelving units, inside cabinets or within display cases—are prone to moisture accumulation and condensation, creating favourable microhabitats for fungal activation [68]. Conversely, excessive or poorly directed airflow may facilitate spore dispersal and cross-contamination between objects, highlighting the need for carefully balanced ventilation strategies tailored to specific spatial configurations [68].

Routine maintenance represents an essential component of HVAC-based preventive conservation. Dust and organic residues can accumulate within ducts, filters and coils, forming secondary reservoirs of fungal propagules that may be re-aerosolised during system operation [68]. Under climate-change scenarios characterised by increased thermal stress and energy demand, the likelihood of HVAC interruptions and performance fluctuations is expected to rise, further underscoring the importance of resilient system design and rapid-response monitoring protocols [63].

### 5.3. Dust and Spore Ecology in Indoor Environments

Dust in indoor heritage environments represents an active ecological matrix rather than a passive contaminant. A substantial fraction of settled dust consists of organic matter—including textile fibres, skin flakes and residues of conservation materials—that provides readily available nutrients for fungal growth when moisture becomes available [69]. As a result, dust layers can function as temporary microbial reservoirs, supporting rapid fungal activation following transient humidification events.

Airborne fungal spores constitute a persistent and dynamic component of indoor aerobiology. Conidia of *Aspergillus*, *Penicillium* and *Cladosporium* retain viability for prolonged periods and may germinate rapidly once exposed to favourable microclimatic conditions [70]. Indoor spore loads are influenced by multiple factors, including HVAC operation, outdoor air exchange, visitor movement and cleaning practices, which together determine patterns of deposition and resuspension [71].

Periodic rehydration of dust deposits—caused by night-time humidity increases, condensation episodes or short-term HVAC fluctuations—can temporarily transform dust-covered surfaces into microbial hotspots [69]. In such conditions, dust acts simultaneously as a nutrient source and as a physical scaffold facilitating hyphal development and surface colonisation. These processes are particularly relevant in storage areas, on shelving systems and within textile collections, where dust accumulation is often heterogeneous and difficult to detect.

From a preventive conservation standpoint, controlling dust accumulation and airborne spore concentrations is therefore integral to mitigating fungal biodeterioration risk. Effective strategies include high-efficiency air filtration, regular and targeted cleaning protocols, and the management of visitor-induced particulate disturbance [69,70,71].

As climate-driven increases in indoor temperature and humidity variability become more frequent, dust-mediated fungal activation is expected to play an increasingly significant role in biodeterioration dynamics within heritage collections.

### 5.4. Climate-Risk Projection Tools for Preventive Conservation

Climate-risk projection tools have become increasingly important for anticipatory management of fungal biodeterioration in cultural heritage collections. These frameworks integrate climatic projections, environmental monitoring data and material vulnerability assessments to identify future risk scenarios and prioritise preventive interventions [72,73].

By shifting the focus from post-damage restoration to foresight-based planning, such tools support more resilient conservation strategies under conditions of increasing climatic variability.

The STRENCH (Strengthening Resilience of Cultural Heritage at Risk) methodology employs spatial modelling and climate indicators to predict areas and collections most susceptible to climate-induced deterioration. Relative humidity, temperature variability and extreme moisture events are central parameters within this framework, reflecting their strong influence on fungal physiology and activation dynamics [72].

Similarly, the HERACLES system combines climate exposure analysis with building vulnerability assessment to enhance preparedness for climate-related hazards affecting heritage environments [73]. A key strength of climate-risk projection tools lies in their capacity to bridge global and regional climate scenarios with local indoor microclimatic conditions.

By integrating downscaled climate data with hygrothermal simulations and object-level monitoring, these models enable the identification of concealed risk hotspots within buildings, such as storage areas, display cases and structural interfaces where moisture accumulation is likely to occur [65,74,75,76,77].

From a preventive conservation perspective, the application of climate-risk projection frameworks facilitates evidence-based decision-making and targeted resource allocation. These tools support the development of early-warning systems, adaptive climate-management strategies and long-term resilience planning, thereby mitigating fungal biodeterioration before irreversible material damage arises [72,73,74,75,76,77,78,79,80].

## 6. Damage to Organic Materials

**Figure 3:**
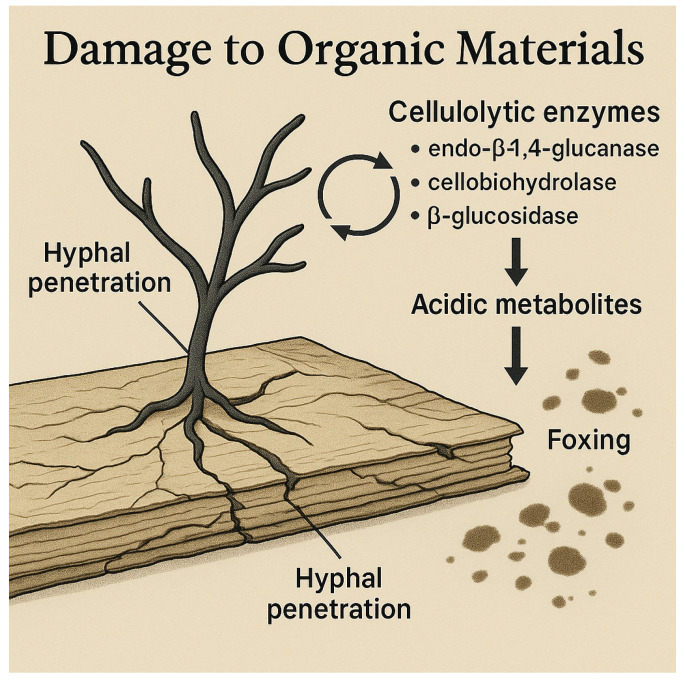
Fungal damage to organic materials: hyphal penetration into paper, production of cellulolytic enzymes, and release of acidic metabolites that cause the discoloration known as foxing.

The chemical and mechanical stability of organic cultural heritage materials is profoundly compromised by the metabolic activity of biodeteriogenic fungi (Figure 3), whose enzymatic systems are specifically adapted to depolymerize cellulose- and protein-based substrates [81,82]. In cellulose-rich materials such as paper, papyri and textile supports, coordinated complexes of extracellular enzymes—including endo-β-1,4-glucanases, cellobiohydrolases and β-glucosidases—progressively cleave the cellulose backbone, leading to fibre weakening, loss of tensile strength and increased fragility [81,82,83].

Microscopic investigations of biodeteriorated manuscripts and archival documents have revealed extensive hyphal penetration within the fibrous network, resulting in microfissures that expand under mechanical stress and recurrent humidity fluctuations [84,85]. These alterations often precede visible damage and may remain undetected during early stages of deterioration. Over time, affected materials exhibit brittleness, powdering, surface erosion and structural deformation, including curling, planar distortion and delamination of surface layers and sizing agents [86]. Fungal biodeterioration is further intensified by the production of secondary metabolites. Organic acids such as oxalic, citric and gluconic acids—commonly produced by *Aspergillus*, *Penicillium* and *Chaetomium* species—promote localised acid hydrolysis of cellulose and destabilisation of inks and pigments, contributing to halo formation, colour bleeding and chemical weakening of media [87,88]. Chromatic alterations, including foxing and dark spotting, are frequently associated with melanin-producing fungi, whose pigments display high chemical stability and strong substrate affinity, rendering discoloration resistant to most conventional conservation treatments [89,90].

Archaeological papyri often show advanced deterioration patterns characterised by fibre erosion, granular disintegration and collapse of the fibrous matrix, particularly in contexts affected by recurrent moisture exposure or elevated relative humidity [91]. Comparable degradation processes have been documented in canvas paintings, where cellulose-based textile supports are treated with carbohydrate-rich consolidants or adhesives that inadvertently increase nutrient availability and stimulate fungal metabolic activity [92].

Overall, fungal damage to organic materials develops as a progressive, cumulative and frequently subclinical process, in which biochemical destabilisation precedes macroscopic alteration by weeks or months [83]. Reliance on visual inspection alone is therefore insufficient for early detection. Effective assessment of fungal damage requires an integrated diagnostic approach combining microstructural analysis, spectroscopic techniques and environmental monitoring to identify early metabolic activity and mitigate irreversible loss of material integrity [93].

### Environmental Drivers and Climate Change Implications

Environmental instability represents one of the most potent accelerators of fungal biodeterioration in cellulose-based heritage materials. Even short-lived excursions in relative humidity above approximately 60% may trigger the activation of dormant fungal propagules, initiating germination and the early stages of enzymatic degradation [94]. Seasonal humidity peaks, transient condensation on cold or poorly ventilated surfaces, and microhabitats characterised by elevated water activity—such as those occurring within fibre networks, binding joints or stratified material layers—create favourable conditions for mycelial penetration and metabolic activation [95].

These microclimatic disturbances not only promote the onset of fungal activity but also intensify its progression. Recurrent RH fluctuations induce cycles of swelling and shrinkage in hygroscopic substrates, exacerbating existing microfissures and facilitating deeper hyphal ingress. Temperature anomalies, particularly those associated with contemporary heatwave events, further enhance fungal metabolic rates and sporulation efficiency, accelerating biochemical depolymerisation, acidogenesis and chromatic alteration [96].

Under conditions of acute moisture exposure, including condensation bursts or surface wetting, fungal colonisation may proceed rapidly and extensively, overwhelming already weakened fibre matrices. Papyri, manuscripts and parchment artefacts are especially vulnerable, as the combination of hygroscopic behaviour, capillary water absorption and reduced structural cohesion allows fungi to establish quickly and compromise material integrity at both microscopic and macroscopic scales [97].

Historic buildings further amplify these risks due to heterogeneous thermal gradients, limited insulation and a strong propensity to develop localised microclimatic niches. Within a single institution, significant variability may occur among storage units, shelving systems and display areas, necessitating spatially resolved monitoring and tailored preventive strategies [98]. Contemporary conservation practice must therefore integrate high temporal and spatial sensitivity in environmental diagnostics to detect early deviations capable of triggering fungal activation.

This evolving scenario aligns with international and European policy frameworks, including the UN 2030 Agenda for Sustainable Development (Target 11.4) and the EU Strategy for Climate Adaptation (COM/2021/82), which explicitly recognise the systemic vulnerability of cultural heritage to climate-induced environmental instability [99]. In this context, modern risk assessment tools—such as the STRENCH methodology—play an indispensable role in preventive conservation by integrating climate foresight, microclimatic modelling and vulnerability analysis into a unified, anticipatory decision-making framework.

## 7. International and European Frameworks for Climate-Resilient Heritage Preservation

The growing recognition of climate-related risks to cultural heritage has progressively reshaped international and European conservation policies. Climate-induced environmental instability—manifesting through increased frequency of extreme events, moisture variability and thermal stress—has highlighted the systemic vulnerability of heritage collections and the limitations of purely reactive conservation approaches [99,100,101].

At the global level, the UN 2030 Agenda for Sustainable Development, particularly Target 11.4, explicitly calls for strengthened efforts to protect and safeguard cultural and natural heritage as a core component of resilient and sustainable societies [100]. Within this framework, cultural heritage preservation is no longer regarded solely as a conservation issue but as an integral element of climate adaptation and risk reduction strategies.

In the European context, the EU Strategy for Climate Adaptation (COM/2021/82) further emphasises the exposure of cultural heritage to climate-driven hazards, including flooding, heatwaves and prolonged humidity anomalies [101]. The strategy promotes the integration of climate risk assessment into heritage management, encouraging the adoption of preventive, evidence-based and forward-looking approaches capable of anticipating future environmental stressors.

Together, these policy frameworks mark a decisive transition from post-damage restoration toward anticipatory and climate-resilient governance of heritage collections. They underscore the importance of environmental monitoring, predictive risk assessment and adaptive management as foundational tools for mitigating climate-related biodeterioration, particularly in indoor environments where microclimatic instability directly influences fungal activity and material vulnerability [99,100,101].

### 7.1. Risk Assessment and Predictive Methodologies

Anticipatory risk assessment has become a central component of contemporary heritage conservation, particularly in relation to fungal biodeterioration, whose activation thresholds and metabolic dynamics respond rapidly to environmental variability [102].

Predictive methodologies increasingly integrate climatic indicators, microclimatic data and material vulnerability parameters to identify collections and environments most susceptible to climate-related deterioration. Relative humidity remains the dominant driver within most risk assessment frameworks, as it directly governs spore germination, hyphal development and enzymatic activation [102].

Temperature instability acts as a synergistic factor, accelerating sporulation and metabolic kinetics, especially among xerophilic and extremotolerant taxa adapted to fluctuating indoor environments [103]. Sudden moisture inputs—caused by flooding, structural leakage or HVAC malfunction—represent acute high-risk scenarios capable of inducing severe biodeterioration of paper- and parchment-based materials within short timeframes [104].

Preventive planning benefits from the analytical use of international standards and institutional guidelines (e.g., ISO and IFLA), which function as benchmark frameworks for evaluating risk severity, vulnerability, and exposure over time. When applied within a risk-based approach, these guidelines enable the prioritization of mitigation measures targeting environmental instability, moisture-related hazards, and biological contamination, thereby strengthening the methodological robustness and reproducibility of preventive conservation strategies.

Predictive modelling platforms such as STRENCH and HERACLES integrate these variables into decision-support systems designed to enhance preparedness and resilience. By combining climate projections, hygrothermal simulations and collection-specific vulnerability assessments, these tools enable the identification of risk hotspots, the prioritisation of monitoring efforts and the design of targeted preventive interventions [72,75,102,103,104,105].

Overall, risk assessment and predictive methodologies provide the methodological foundation for a shift toward proactive, climate-adaptive conservation strategies. Their systematic application supports evidence-based decision-making and facilitates the allocation of resources toward areas of highest vulnerability, thereby mitigating fungal biodeterioration before irreversible material damage occurs [102,103,104,105].

### 7.2. Prevention and Monitoring Strategies for Organic Collections

Preventive conservation represents the most effective and sustainable approach to mitigate fungal biodeterioration in organic heritage collections. Maintaining stable thermo-hygrometric conditions below biological activation thresholds is essential to suppress spore germination, limit enzymatic activity and slow acidogenic reactions that compromise the structural integrity of cellulose- and collagen-based materials [106].

Ventilation and filtration strategies play a complementary role by regulating indoor aerobiology and reducing the continuous introduction and redistribution of fungal propagules. Airborne conidia of *Aspergillus*, *Penicillium* and *Cladosporium* are persistently introduced through HVAC systems, visitor movement and dust resuspension, making high-efficiency filtration and balanced airflow critical components of preventive management [107]. Dust control is equally important, as particulate matter acts both as a nutrient reservoir and as a carrier of viable spores capable of rapid activation following transient humidification.

Because early fungal activity often develops in a subclinical manner, reliance on visual inspection alone is insufficient for timely intervention. Comprehensive monitoring strategies should therefore integrate environmental sensing with microbiological and material diagnostics, including culture-based and molecular analyses, imaging techniques and spectroscopic methods, to detect early metabolic signatures before visible damage occurs [108,109].

Rapid response protocols are crucial following acute moisture events. Water ingress, flooding or condensation episodes require immediate environmental stabilisation, rapid drying and, where appropriate, microbiological assessment to prevent explosive fungal colonisation and irreversible fibre collapse [110]. Material choices in conservation treatments also warrant careful consideration, as organic consolidants and adhesives may inadvertently increase nutrient availability and promote fungal regrowth if not properly evaluated [111].

**Figure 4:**
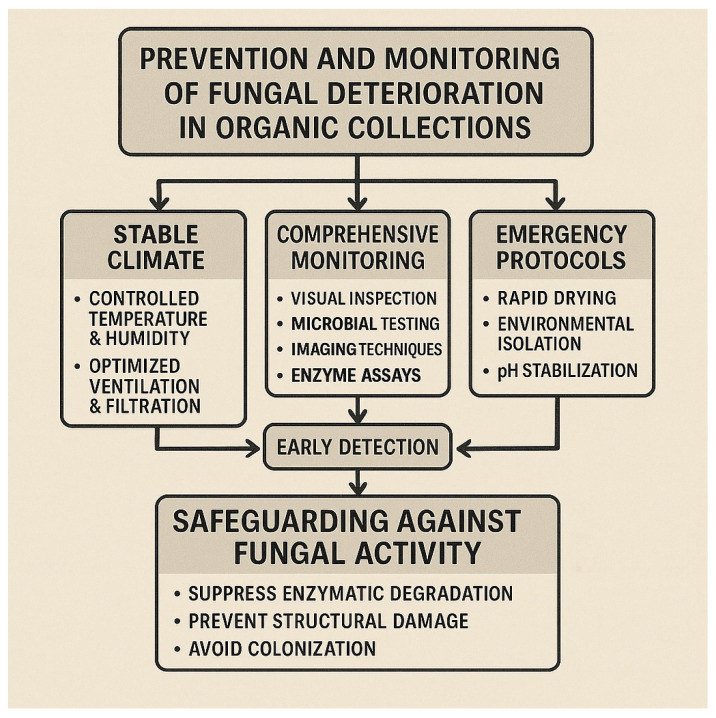
Flowchart outlining key preventive and monitoring strategies for safeguarding organic collections, including stable climate control, comprehensive diagnostics, and rapid emergency response to prevent fungal colonization and enzymatic degradation.

Collectively, these measures form a resilience-oriented preventive framework, in which environmental control, early detection and adaptive response converge to protect vulnerable organic collections from climate-driven biodeterioration. The systematic integration of monitoring data into risk assessment and management strategies is therefore essential for sustaining long-term preservation under increasingly variable climatic conditions (Figure 4). This integrated approach provides the conceptual bridge to the concluding reflections presented in Section 8.

## 8. Conclusions and Future Perspectives

Fungal biodeterioration remains one of the most pervasive, adaptive and climate-sensitive threats to organic cultural heritage. Cellulose- and collagen-based materials—including paper, papyri, parchment and textile supports—provide highly favourable substrates for filamentous fungi whose enzymatic systems progressively compromise both the physical stability and the informational value of heritage objects, often long before visible signs of damage become apparent.

Environmental instability, particularly fluctuations in relative humidity and temperature, is therefore framed as the primary catalyst for fungal activation and metabolic intensification in indoor heritage environments. The increasing frequency of extreme climatic events—such as heatwaves, intense precipitation and moisture ingress—further exacerbates these risks, especially in historic buildings characterised by limited insulation, heterogeneous construction materials and reduced buffering capacity. In such contexts, even short-lived deviations from recommended environmental conditions may initiate irreversible biodeterioration processes.

### 8.1. Future-Oriented Conservation Priorities

Safeguarding organic cultural heritage under evolving climatic conditions requires a decisive transition from reactive restoration toward anticipatory, climate-informed preventive conservation. Predictive risk assessment frameworks, including methodologies such as STRENCH, provide essential tools for identifying future exposure scenarios and prioritising collections most vulnerable to environmental instability.

Equally important is the implementation of continuous, high-resolution microclimatic monitoring systems capable of maintaining environmental conditions below biological activation thresholds. When combined with early-warning approaches integrating environmental, microbiological and material indicators, such systems enable timely intervention before fungal activity progresses to advanced stages of deterioration.

Material awareness must also inform future conservation strategies. The selection of consolidants, adhesives and repair materials should minimise the introduction of nutrient-rich compounds that may inadvertently promote fungal colonisation under favourable microclimatic conditions. Addressing these challenges effectively will depend on sustained interdisciplinary collaboration among conservation scientists, microbiologists, materials engineers and climate-policy specialists.

### 8.2. Critical Research Gaps

Despite substantial advances, key research gaps persist. The long-term behaviour and ecological dynamics of xerophilic and extremotolerant fungi increasingly detected in heritage repositories remain insufficiently understood. Further molecular-level investigation into enzyme–substrate interactions under fluctuating environmental regimes is needed to improve predictive models of biodeterioration.

In parallel, the development and validation of low-toxicity, sustainable biocontrol strategies suitable for indoor heritage contexts represent a critical research priority. Integrating global and regional climate projections with high-resolution indoor microclimate modelling also remains a significant methodological challenge, yet one that is essential for advancing climate-resilient conservation paradigms.

Addressing these unresolved issues will be fundamental to the development of predictive, material-sensitive and climate-adaptive conservation strategies capable of safeguarding organic cultural heritage throughout the twenty-first century (Table 3).

### 8.3. Final Remark

Safeguarding the integrity of cultural memory embodied in cellulose-based artifacts transcends technical preservation; it constitutes a profound commitment to intergenerational responsibility. By embracing proactive, climate-adaptive conservation strategies—rooted in foresight, scientific rigor and collaborative governance—we ensure that the documentary and artistic heritage entrusted to us remains authentic, accessible and meaningful for the generations that follow.

## Figures and Tables

**Table 1:** Material-specific vulnerabilities and dominant biodeteriogenic fungi in cultural heritage substrates.

Material	Main Composition	Predominant Biodeteriogenic Fungi	Main Degradation Mechanisms	Typical Signs of Deterioration	Representative References
Paper (books, manuscripts, archival documents)	Cellulose, hemicelluloses; sizing agents and fillers	*Chaetomium globosum*; *Trichoderma* spp.; *Aspergillus* spp.; *Penicillium* spp.	Cellulolysis, acidification, hyphal penetration of fibre network	Loss of mechanical strength, brittleness, foxing, surface erosion, powdering	[9,42,43,44,45,48]
Papyrus	Cellulose-rich plant fibres with residual polysaccharides, mineral inclusions and salts	*Chaetomium* spp.; *Aspergillus* spp.; halotolerant and xerotolerant fungi	Cellulose depolymerisation, salt–moisture interactions, fibre fragmentation	Granular disintegration, fibre collapse, dark spotting, extreme fragility	[3,42,45]
Plant-derived textiles (linen, cotton, hemp)	Cellulose fibres	*Cladosporium* spp.; *Penicillium* spp. *Aspergillus* spp.	Cellulolytic degradation, pigment production, deep fibre penetration	Weakening of weave, discoloration, musty odours, tearing	[43,44,45]
Animal-derived textiles (wool, silk)	Protein fibres (keratin, fibroin)	*Aspergillus* spp.; *Penicillium* spp.; *Eurotium* spp.	Proteolysis, fibre embrittlement, moisture-induced deformation	Loss of elasticity, fibre breakage, surface powdering	[7,43,44]
Parchment and leather	Denatured collagen	*Aspergillus* spp.; *Eurotium* spp.; *Engyodontium* spp.	Proteolysis, gelatinisation, acidification, moisture uptake	Curling, delamination, tacky surfaces, loss of mechanical integrity	[7,18,43]
Canvas-based paintings and polymaterial artworks	Textile support (linen or cotton) combined with ground layers, binders, pigments and varnishes	*Cladosporium* spp.; *Paecilomyces* spp.; *Penicillium* spp.	Cellulolytic and enzymatic degradation of supports and binders, pigment alteration	Chromatic staining, weakening of canvas, detachment of paint layers	[8,43,44,45]
Consolidated or treated organic materials	Organic polymers, adhesives, consolidants applied during restoration	Xerophilic and opportunistic fungi (*Wallemia sebi*; *Aspergillus restrictus*)	Use of conservation materials as nutrient sources, growth at low water activity	Recurrent fungal growth after treatment, surface dulling, widespread staining	[5,9,37]

**Table 2:** Critical climate thresholds for fungal deterioration of organic heritage.

Climate Driver/Condition	Approximate Critical Threshold	Principal Fungal Response	Most Vulnerable Organic Materials	Implications for Preventive Conservation	Representative References
Sustained high relative humidity	≥60–65% RH for ≥2–3 days	Activation of dormant conidia, onset of germination	Paper, papyri, archival documents, parchment, textiles	Avoid RH excursions above ~60%; correct leaks rapidly	[57,58,59]
Very high relative humidity/near-saturation	80–95% RH	Rapid mycelial growth, strong enzymatic activity	All hygroscopic organic supports	Strict upper RH limits; robust dehumidification	[59,60,61,62,63]
Surface condensation and micro-wetting	Local RH ≈ 100%	Localised germination hotspots	Paper near exterior walls, framed works	Mitigate thermal bridges; improve air circulation	[60]
Water activity (aw) in materials	aw ≥ 0.70 (xerophiles ≈ 0.65)	Metabolic activation at lower bulk RH	Paper, papyri, historic canvases	Use aw-based risk models, monitor layered systems	[64,65,66]
Repeated RH fluctuations	Cycles crossing 60–80% RH	Swelling–shrinkage stress; deeper penetration	Parchment, papyri, sized papers	Limit amplitude/frequency of RH swings	[58,65,66]
Elevated temperature at high RH	≥25–30 °C with RH > 60%	Enhanced enzymatic kinetics, faster growth	Cellulosic and proteinaceous materials	Consider combined T–RH effects; heatwave risk	[63,66]
Acute moisture events	Free water or capillary wetting	Explosive colonisation; fibre collapse	Manuscripts, papyri, leather, canvas	Emergency drying, isolation, microbiological assessment	[60,65]
High dust load and airborne spores	Persistent particulate deposition	Dust as nutrient reservoir and spore carrier	Archives, libraries, textiles	Filtration (HEPA/MERV 13–16), dust management	[61,62]
HVAC malfunction	Uncontrolled RH/T drift; shutdowns	Recurrent threshold crossing; regrowth cycles	Collections in historic buildings	Resilient HVAC design; alarms; rapid response	[63]

**Table 3:** Summary of Key Environmental and Conservation Factors Influencing Fungal Biodeterioration.

Category	Key Factors	Effects on Heritage Materials	Representative References
Environmental Drivers	High RH; temperature fluctuations; condensation; elevated aw; dust load; airborne spores; HVAC malfunction	Activation of dormant spores; accelerated enzymatic degradation; hyphal penetration; microfissuring; chromatic alteration	[67,68,69,70,71,72,73]
Material Vulnerability	Hygroscopic substrates; organic consolidants/adhesives; layered microstructures	Moisture absorption; structural weakening; nutrient availability; increased susceptibility	[69,70,71,74,75,76,77]
Fungal Mechanisms	Cellulolytic/proteolytic enzymes; acidogenesis; pigment production; fibre penetration	Depolymerization; fibre fragmentation; acid hydrolysis; staining; loss of strength	[81,82,83,84,85,86,87,88,89,90]
Climate Change Impacts	Heatwaves; storm moisture; unstable microclimates; xerophilic fungi	More outbreaks; reduced effectiveness of controls	[78,79,80]
Monitoring Strategies	Visual inspection; culture; molecular assays; SEM; FTIR; enzyme tests; environmental sensing	Early detection; improved risk assessment	[108,109]
Preventive Measures	Climate control; ventilation/filtration; dust management; adaptive planning	Reduced activation; suppressed degradation; improved resilience	[67,106,107,108,109,110,111]
Risk-Assessment Tools	STRENCH; HERACLES; climate projections; microclimate modelling	Predict vulnerability; identify hotspots; inform interventions	[72,73,74,75,76,77,102,103,104,105]

## Data Availability

No new data were created or analyzed in this study.

## Abbreviations

The following abbreviations are used in this manuscript:

COM (2021) 82 final	EU Strategy for Climate Adaptation
FTIR	Fourier Transform Infrared Spectroscopy
HEPA	High-Efficiency Particulate Air
HERACLES	HEritage Resilience Against CLimate Events on Site
HVAV	Heating, Ventilation, and Air Conditioning
IFLA	International Federation of Library Associations and Institutions
ISO	International Organization for Standardization
MERV	Minimum Efficiency Reporting Value
PICOS framework	Population-Intervention-Comparison-Outcomes-Study-Design
PRISMA 2020	Preferred Reporting Items for Systematic Reviews and Meta-Analyses (2020)
SEM	Scanning Electron Microscopy
STRENCH	Strengthening Resilience of Cultural Heritage at Risk
UV	Ultraviolet
